# The History of the Cholera in Exeter in 1832

**Published:** 1850-04

**Authors:** 


*The History of the Cholera in Exeter in* 1832. By Thomas Shapter, M. D., Physician to the Devon and Exeter Hospital, &c. 8vo. pp. 297. London, 1849.

This account of the first epidemic of cholera in Exeter includes many details, which, although of historical interest to the people of that city, do not require special notice here. Notwithstanding the disease had given timely warning of its approach, and a panic fear of it existed throughout the community, there were but few effectual measures of precaution adopted, before it actually arrived. Thus, the multiplicity of nuisances, the want of underground drainage, the insufficient supply of water, the want of hospitals for the sick and suitable places of interment for the dead, were discovered too late to be remedied, with any prospect of arresting the progress of the disease. The natural position of Exeter is particularly salubrious. It is situated upon the side of a wedge-shaped hill, with a broad river flowing at its foot, and enjoys a mild and genial climate; but at the same time it is a very old city, closely and irregularly built, with narrow streets and projecting upper stories. Nuisances of the most offensive kind, we are told by Dr. Shapter, had been allowed to accumulate for years, and even with the daily expectation of the approach of the cholera, many of the inhabitants objected strongly to the removal of such of them as were a source of profit. The disease prevailed most extensively in the older part of the city; that portion, which, from the lowness of its situation, the closeness and filth of its streets and houses, and the misery of its inhabitants, was most likely to favor its spread. The great prevalence of the disease in those quarters bordering upon the river, and the comparative immunity enjoyed by the higher and more open modern town and suburbs, are admirably shown by a map, accompanying Dr. Shapter's book, in which the spots visited by the cholera are marked by red lines; these form uninterrupted rows in the lower and thickly populated portion of the city, but are few and scattered, along the wider and more airy streets. It appears that some hope had been entertained by the authorities of Exeter, that the disease might be excluded by a strict enforcement of quarantine regulations; but while its entrance into the port was thus hindered, it introduced itself into the city by other unguarded avenues. The first cases occurred in persons who had arrived respectively from London and Plymouth, in both of which places the cholera was prevailing. One had been suffering for some days from the premonitory diarrhœa, the other had nursed a patient who had died of the cholera. During the first week, after the occurrence of these cases, it did not diffuse itself to any great extent, a few cases only occurring daily; "but from this time to the commencement of the next fortnight, it rose rapidly and continuously to its height, then gradually subsided, and in about a month afterwards, had, as a prevailing epidemic, passed away." The population of Exeter was at this time about 28,000. The total number of cases from the 19th of July to the 27th of October, was 1,135, and the deaths during that period, 345. "The whole of the deaths," Dr. Shapter says, "were not returned, as will subsequently be shown, and the daily numerical returns record many deaths, not previously reported as cases," but we cannot find any correction afterwards of these errors. Every one is aware of the sources of error in cholera statistics, and although when these are collected from different and numerous sources, it may be assumed, that the errors in one report neutralize those in another, yet taken singly, they offer a very fallacious basis for induction, and particularly as to the rate of mortality. But a full consideration of this important subject would lead us into too large a digression, we therefore return to the general results deduced by Dr. Shapter from his statistics. The general impression, that, as the disease numerically declined, its malignancy or fatality diminished in like proportion, is not confirmed by his observation. He finds that the rate of mortality, through the whole course of the epidemic, was constantly about 35 per cent. Nor is it true, according to these results, that other diseases subsided as the cholera advanced. Excluding the few deaths, which occurred after the 19th of September, the deaths from cholera were 336, those from other causes 90, or 45 per month, the average mortality in other years being about 50 monthly, so that the deaths from cholera seem merely to have been superadded to the ordinary mortality. We are not informed, how many of these other cases were due to bowel diseases. The influence of age, appears from a carefully constructed table, to be, that "during infancy, the mortality is rather above that which takes place between the ages of 10 and 35, when the deaths are by no means numerous, and that, after the age of 35, there is a constant increase in the relative amount of mortality, but, that its greatest proportionate amount takes place after 55 years of age." Hence the mortality is the greatest at the two extremes of life. With regard to the mode of propagation of epidemic cholera, Dr. Shapter states, that after a full consideration of the controversy, he has "arrived at the conclusion, that the Asiatic cholera is essentially an epidemic, originating in and chiefly due to aërial influences, but capable, under peculiar and rare conditions, of being transmitted from man to man." We feel inclined to pass by speculations upon the origin and nature of cholera, of which we have had lately a sufficient quantity, but Dr. Shapter alludes here to one fact which is of particular importance in a practical point of view, viz: that the collapse is not due to the loss of fluid by the intestinal canal, for in some instances, and those indeed usually the most rapid and fatal, there was no diarrhœa of consequence. This fact is not satisfactorily explained, by alleging that the liquid effusion is, in these instances, retained in the bowels, as this is never so abundant as to account for the collapse. We venture to assert, that in many cases of watery diarrhœa there is more liquid poured out from the bowels than in many of the most malignant cholera. Dr. Shapter, indeed, goes so far, as to advance, that the choleraic discharges may be beneficial by an elimination of the "*materies morbi*," a supposition, which might now be very consistently maintained by the believers in the "*cholera fungi*." The nervous system, or that particular portion of it termed the sympathetic system, is considered by our author, to be the portion of the economy which receives the first impression of the epidemic influence. All the prominent phenomena of the disease can be beautifully explained by this theory, and there may be very good reason to believe, from what we know of the functions of the sympathetic system, that it is implicated seriously in the attack; but, however ingenious the supposition, it is a mere diversion of the mind, having no practical bearing. For whether it be the nerves or the blood-vessels, or the blood itself, or the bowels, which are first "disordered," we need not stop to inquire; the sick man calls for aid, and happy is he who can resolutely say, *this medicine* has cured another, it *may* save you! Certainly, if there is any disease, before which the impotence of theories, as therapeutical indications, is demonstrated, that disease is the Asiatic cholera. It will not be necessary for us to extract Dr. Shapter's account of the general treatment pursued in Exeter; it is what is called a rational one, because it holds fast to the results of the "*juvantia et lœdentia*" in other morbid states, which offer a greater or less similitude to the cholera.

We will therefore close this brief and imperfect notice of a work, which is evidently the result of a great deal of laborious perseverance, by recommending its perusal to all those who are desirous of collecting historical facts, in reference to a subject of so much interest as that of epidemic cholera; since it contains, besides the points which we have noticed, much that is deserving of attention even from the unprofessional reader. M. S.

